# Effect of Nurses' Work Experiences in a COVID-19 Unit on Depression: Mediation Effect of Resilience and Moderated Mediation Effect of Organizational Trust

**DOI:** 10.3389/fpubh.2022.897506

**Published:** 2022-08-04

**Authors:** Eun-Young Doo, Sujin Choi

**Affiliations:** ^1^Nursing Department, Myongji Hospital, Goyang-si, South Korea; ^2^College of Nursing, Woosuk University, Jeonju, South Korea

**Keywords:** COVID-19 infection, depression, organizational trust, nurses, resilience

## Abstract

**Aim:**

Nurses work with a lack of organizational support and perceive an unsafe environment from their organizations, which has been related to depression. This study aimed to investigate the effect of nurses' work experiences in a COVID-19 unit on their depression, the mediation effect of resilience, and the moderated mediation effect of organizational trust.

**Methods:**

Participants were 132 nurses working at a general hospital. Through questionnaires, data were collected during the COVID-19 pandemic and analyzed using SPSS 25.0 and SPSS PROCESS macro.

**Results:**

Mean resilience was 2.15 ± 0.76; mean organizational trust was 3.03 ± 0.74; mean depression was 0.76 ± 0.63. 65.6% nurses with work experience in a COVID-19 unit had depression. Of the 27.2% nurses who showed moderate or higher levels of depression. Nurses' work experiences in a COVID-19 unit had a direct effect on depression and indirectly affected depression via resilience as a mediator. Resilience had a partial mediation effect, and organizational trust had a significant moderated mediation effect in the path from nurses' work experiences in a COVID-19 unit to depression mediated by resilience.

**Conclusions:**

This study emphasizes the key role that healthcare organizations play in providing sufficient support to nursing staff to protect them from depression by improving nurses' resilience and organizational trust during the pandemic. Healthcare organizations need to develop a systematic structure to provide organizational support to nurses so that the organizational trust and resilience of nurses can be maximized.

## Introduction

The COVID-19 outbreak has increased the threat to the psychological health of nurses. Research indicates that the greatest challenges for hospital leaders are managing COVID-19 while keeping the staff safe (1) and maintaining staffing (2). Nurses have worked with a lack of personal protective equipment (PPE) (2) and organizational support (3), which causes them concerns regarding getting infected and transmitting the virus to their family (4). These concerns are accompanied by depression (5). The symptoms of depression increase the risk of suicide (6); the suicide rate among nurses is 23%, which is higher than the national average (7). Hence, a study on depression among nurses during the COVID-19 pandemic was necessary.

Owing to the efforts of healthcare providers, the treatment of patients affected by the COVID-19 pandemic has been managed well in South Korea. A recent study of factors in the work intention of hospital workers revealed that, while 60% accepted the nature of their work, 54.4% reported that their job was dangerous (8). As in other countries, nurses are on the frontline of healthcare services (9). Compared to other workers, nurses were re-ported to be most likely to quit or to have the intention to quit (10). They also reported a lack of material resources, physical stress, psychological stress, and depression arising from the possibility of transmitting the virus to others (5). The resilience of nurses in such extreme circumstances has been tested.

The American Nurses Association (11) provided guidelines and information related to COVID-19 to support the mental health and resilience of nurses. Resilience refers to an individual's ability to deal with challenging circumstances, thereby mitigating the negative impact of distressing events and reducing the rate of posttraumatic stress disorders (12). According to Rubin et al. (13), people who have experienced traumatic events and exhibited resilience do not experience negative psychological symptoms. However, the expectation that nurses will have a higher level of resilience as an individual trait is to misunderstand the original intention of resilience (9, 14). During traumatic events, nurses may feel that if they do not deal with the situation then they are not resilient enough, and it is their fault (9). Such a feeling could mean that nurses are in danger of experiencing psychological symptoms from their work during the COVID-19 pandemic and the expectation that nurses will be resilient. Thus, the focus should shift from individual nurses to adequate and trustworthy organizational support for nurses to ensure their resilience.

According to previous studies that adopt an organizational approach toward employees, organizational trust can be considered an informal agreement between employees and their organizations or managers (15). Organizational trust enhances the organizational commitment of employees (16). However, during public health emergencies, nurses experienced an “unsafe environment and fear of abandonment” within their organizations (3), which has been related to psychological stress and depression. Consequently, there is a need to ensure organizational trust among nurses in South Korea in relation to their work experiences and to address their resilience and depression during this pandemic.

Therefore, this study endeavored to investigate the relationships between work experiences, depression, resilience, and organizational trust among Korean nurses during the COVID-19 pandemic.

### Purpose

This study aimed to (1) examine the mediation effect of resilience on the relationship between nurses' work experiences in a COVID-19 unit and depression and (2) verify the moderated mediation effect of organizational trust on the relationship between nurses' work experiences in a COVID-19 unit and depression, with resilience as a mediating variable.

## Methods

### Design

This study used a cross-sectional survey and tested a hypothetical model to examine the mediation effect of resilience and the moderated mediation effect of organizational trust on the relationship between nurses' work experiences in a COVID-19 unit and depression.

### Study Sample

The participants in the study were clinical nurses at a general hospital with more than 500 beds in South Korea. As a nationally designated infection control institution, the general hospital operated COVID-19 units which included five intensive care units, one designated unit, and 33 pneumonia surveillance unit beds. The hospital had admitted 47 patients with a diagnosis of COVID-19 since January 26, 2020. The average number of patients hospitalized on a daily basis was 5.26. Approximately 31.9% of the patients needed care in the intensive care unit, and 23.4% of the patients needed ventilator therapy. The fatality rate was ~17.0% (17). At the time of the study, the number of confirmed COVID-19 cases in South Korea was 12,484 and the death toll was 281, with a mortality rate of 2.25% (18). Nurses who worked for COVID-19 patients were selected in the existing general unit and intensive care unit. They could request to rotate to a general unit (19).

A convenience sampling method was used in this study. The specific inclusion criteria for the subjects were: (1) nurses who had been working in a ward or intensive care unit for at least 1 year and (2) nurses who had directly cared for confirmed or suspected patients with COVID-19 for at least 1 month. The suspected patients with COVID-19 were those who had respiratory symptoms and fever or those who were in self-isolation. The exclusion criteria were: (1) nurses working in departments other than wards and (2) nurses who did not directly care for patients.

According to Mitchell (20), a sample size of 10 to 20 times the number of independent variables is needed to minimize sampling errors and validate a model. A sample size of 110–220 was required, given that there were 11 observed variables in this study. The total number of participants in this study was 132, satisfying the minimum requirement for sample size.

### Instruments

This study used a structured questionnaire consisting of 31 questions (8 for general characteristics, 10 for resilience, 4 for organizational trust, and 9 for depression). The general characteristics included age, gender, marital status, education, working units, total clinical experience, experience of working in a COVID-19 unit, and willingness to apply to the COVID-19 unit in the future. The instruments in this study were used after permission was obtained from the original author, except for the instrument for depression, which was distributed free of charge on a website.

#### Nurses' Work Experiences in a COVID-19 Unit

The nurses' experience of working in a COVID-19 unit was assessed through an item from general characteristics: whether they had worked in a COVID-19 unit or had provided care to suspected patients for more than 1 month.

#### Organizational Trust

Nurses' organizational trust was assessed using an instrument of organizational trust developed by Nyhan and Marlowe (21) and translated into Korean by Hong (22). Hong (22) excluded a subscale regarding trust in the supervisor from the original instrument. The instrument for organizational trust consisted of four items. Participants were asked to rate each item on a five-point Likert scale (from 5 = strongly agree to 1 = strongly disagree). The minimum and maximum possible scores were 4 and 20, respectively, with higher scores indicating a higher level of organizational trust among the nurses. Cronbach's alpha was 0.92 in Nyhan and Marlowe's (21) study, 0.79 in Hong's (22) study, and 0.88 in the present study.

#### Resilience

Resilience was assessed by a score measured using an instrument developed by Connor and Davison (23), shortened by Campbell-Sills and Stein (24), and then validated in the Korean context by Jung et al. (25). The instrument consisted of 10 items. Each item was evaluated on a five-point Likert scale (from 0 = not at all to 4 = almost always). Higher scores indicate higher resilience. Cronbach's alpha was 0.89 in Connor and Davidson's (23) study, 0.85 in Campbell-Sills and Stein's (24) study, 0.96 in the study by Jung et al. (25), and 0.94 in the present study.

#### Depression

Depression was measured using the Korean version of the depression screening instrument developed by Spitzer et al. (26) and validated in the Korean context by Park et al. (27). The instrument consists of nine items. The participants were asked to report the frequency with which they had suffered from various symptoms during the preceding 2 weeks. Each item was scored using a four-point Likert scale (from 0 = never to 3 = almost every day), with a total score of 5 or more indicating “depression” of some degree. A total score of 0–4 indicated “normal,” 5–9 indicated “mild depression,” 10–14 indicated “moderate depression,” 15–19 indicated “moderately severe depression,” and ≥20 indicated “severe depression.” Higher scores indicated higher levels of depression. Cronbach's alpha was 0.89 in the study by Spitzer et al. (26), 0.84 in the study by Park et al. (27), and 0.90 in the present study.

### Data Collection and Ethical Considerations

After approval by the Institutional Review Board, data were collected from October 5 to October 20, 2020, the second wave of COVID-19 pandemic in South Korea. Permission from the nursing department of the hospital was also required. Thus, the first author, who was a member of the nursing staff, visited the nursing department of the study hospital and explained the purpose of the study. After this, man-agers of the nursing unit explained the purpose, methods, and inclusion and exclusion criteria of participants to the nurses. In addition, the nurses were advised that they could stop participating in the study at any time, that anonymity would be guaranteed, and that an online questionnaire would be provided. A message about the study was sent to the nurses who voluntarily participated. Consent was obtained from the participants before they began the online questionnaire. Of the 140 questionnaires distributed, 132 questionnaires (response rate of 94.3%) were used for analysis, excluding 8 due to incomplete responses with missing data.

### Data Analysis

The data were analyzed using SPSS WIN 25.0 program and SPSS PROCESS macro (3.1 version). Descriptive statistics were used to analyze the general characteristics of the nurses and the major variables. Pearson's correlation coefficient was used to analyze correlations between variables. When examining correlations in this study, among the general characteristics, age showed a significant positive correlation with resilience and was used as a control variable. The bootstrapping method in SPSS PROCESS macro (Model 7) (28) was used to investigate the moderated mediation effect of organizational trust in the relationship between nurses' work experiences in a COVID-19 unit and depression, with resilience as a mediator.

## Results

### Sample Characteristics

Of the participants, 129 (97.7%) were women, with an average age of 28.86 ± 5.11 years; 115 (87.1%) were unmarried. In addition, 102 (77.3%) had a bachelor's degree or higher, and 96 (72.7%) were working in nursing units. The average total clinical career was 5.74 ± 4.60 years. In total, 64 (48.5%) participants had worked in a COVID-19 unit, and 100 (75.8%) participants said that they would not be willing to apply to a COVID-19 unit in the future.

### Descriptive Statistics and Correlations of Variables

Among the 64 participants who had worked in a COVID-19 unit, mean resilience was 2.15 ± 0.76 out of 4, mean organizational trust was 3.03 ± 0.74 out of 5, and mean depression was 0.76 ± 0.63 out of 3. In total, 54.5% of the nurses had depression, and there was a statistically significant difference in incidence of depression (χ^2^ = 11.611, *p* = 0.016) depending on the experience of working in a COVID-19 unit; 42 (65.6%) nurses with work experience in a COVID-19 unit had depression, while 30 (44.1%) nurses with no experience in a COVID-19 unit had depression. In addition, when examining the severity of depression, of the 36 (27.2%) nurses who showed moderate or higher levels of depression, 23 (35.9%) were nurses with work experience in a COVID-19 unit, and 13 (19.1%) were nurses with no work experience in a COVID-19 unit (Table 1).

**Table 1:** Levels of depression in participants (*N* = 132).

**Level of depression** **severity, score**	**Nurses' work experiences in a COVID-19 unit**						**χ^2^**	***p***
	**Total (*****N*** **=** **132)**		**Yes (*****N*** **=** **64)**		**No (*****N*** **=** **68)**			
	***n***	**%**	***n***	**%**	***n***	**%**		
Normal, 0 to 4	60	45.5	22	34.4	38	55.9	11.611	0.016
Mild, 5 to 9	36	27.3	19	29.7	17	25.0		
Moderate, 10 to 14	23	17.4	12	18.7	11	16.2		
Moderately severe, 15 to 19	7	5.3	5	7.8	2	2.9		
Severe, 20 to 27	6	4.5	6	9.4	0	0		

Resilience was negatively correlated with nurses' work experiences in a COVID-19 unit (r = −0.177, *p* = 0.043). Depression was positively correlated with nurses' work experiences in a COVID-19 unit (r = 0.282, *p* = 0.001) and negatively correlated with resilience (r = −0.449, *p* = 0.000). Organizational trust was positively correlated with resilience (r = 0.227, *p* = 0.009) and negatively correlated with depression (r = −0.186, *p* = 0.033), but had no significant correlation with nurses' work experiences in a COVID-19 unit (r = −0.109, *p* = 0.214). Age was positively correlated with resilience (r = 0.235, *p* = 0.007) and nurses' work experiences in a COVID-19 unit (r = −0.016, *p* = 0.858), and it had no significant correlation with depression (r = −0.107, *p* = 0.224) or organizational trust (r = −0.032, *p* = 0.715).

### The Mediation Effect of Resilience in the Relationship Between Work Experiences in a Covid-19 Unit and Depression

**Figure 1:**
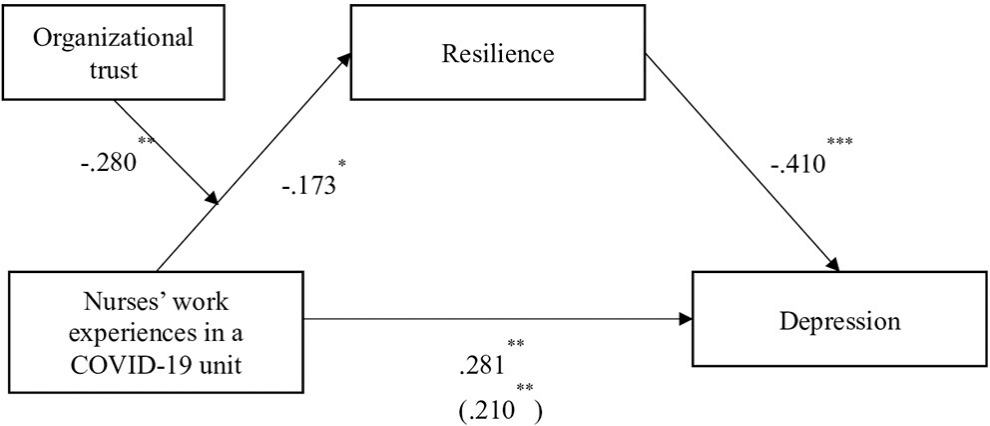
Interaction effect of nurses' work experiences in a COVID-19 unit and organizational trust on resilience.

The participants' work experiences in a COVID-19 unit had a significant negative influence on resilience (B = −0.26, *p* = 0.042) and a significant positive influence on depression (B = 0.35, *p* = 0.001). Participants' resilience had a significant negative influence on depression (B = −0.34, *p* < 0.001) (Table 2). The mediation effect of resilience in the relationship be-tween work experiences in a COVID-19 unit and depression was identified using the PROCESS macro for bootstrapping (Model 7) (28). The mediating effect of resilience on the relationship between nurses' COVID-19 unit work experiences and depression was −0.41, which was significant because the bias-corrected bootstrap confidence interval (CI; −0.47, −0.21) did not contain 0 (Figure 1; Table 2). In addition, work experiences in a COVID-19 unit had a direct influence of 0.21 on depression and an indirect influence of 0.28 on depression (Figure 1). This indicates that the indirect effect of nurses' work experiences in a COVID-19 unit on depression via resilience was greater than the direct effect of nurses' work experiences in a COVID-19 unit on depression. Therefore, this study model showed a partial mediating effect of resilience on the effect of nurses' work experiences in a COVID-19 unit on depression (Figure 1).

**Table 2:** Unstandardized regression coefficients estimating resilience and depression (*N* = 132).

**Predictors**	**Resilience (M)**				**Depression (Y)**			
	**Coeff**.	**SE**	**t**	**95% CI**	**Coeff**.	**SE**	**t**	**95% CI**
Nurses' work experiences in a COVID-19 unit (X)	−0.26	0.13	−2.06*	−0.48, 0.01	0.35	0.11	3.34**	0.07, 0.46
Resilience (M)					−0.34	0.07	−5.11***	−0.47, −0.21
Organizational trust (W)	0.21	0.08	2.59*	0.04, 0.37				
X × W	−0.58	0.16	−3.55**	−0.89, −0.26				
Constant	1.05	0.34	3.05***	0.37, 1.73	1.52	0.29	5.25***	0.95, 2.09
	*R*^2^ = 0.211				*R*^2^ = 0.244			
	*F* = 8.466, *p* = 0.001				*F* = 13.769, *p* <0.001			

CI, confidence interval; Coeff., coefficient; SE, standard error; M, mediator; W, moderator; X, independent variable; Y, dependent variable.

Adjusted for age:

^*^p < 0.05;

^**^p < 0.01;

^***^p < 0.001.

### The Moderated Mediation Effect of Organizational Trust in the Path From Nurses' Work Experiences in a COVID-19 Unit to Depression Mediated by Resilience

Organizational trust had a significant positive effect on resilience (B = 0.21, *p* = 0.01), but had no significant direct effect on depression. The moderated effect of organizational trust between nurses' work experiences in a COVID-19 unit and resilience was significant (B = −0.58, *p* = 0.001) (Table 2). The moderated mediating effect of organizational trust (β = −0.280, *p* < 0.01) was significant only in the relationship between nurses' work experiences in a COVID-19 unit and resilience. The moderated mediating effect index was 0.198. The moderated mediating effect was significant because the bias-corrected bootstrap CI (0.051, 0.334) did not contain 0 (Table 3).

**Table 3:** Indirect effect of nurses' work experiences in a COVID-19 unit on depression and moderated mediation of organizational trust (*N* = 132).

**Nurses' work experience in COVID-19 unit** ** → resilience**		
Condition (level of moderator— organizational trust)	Indirect effect (SE)	Boot 95% CI
Low organizational trust (Mean – 1 SD)	0.198 (0.169)	−0.137, 0.533
Average organizational trust (Mean)	−0.230 (0.120)	−0.467, 0.007
High organizational trust (Mean + 1 SD)	−0.659 (0.171)	−0.997, −0.320
**Nurses' work experiences in a COVID-19 unit** ** → resilience** ** → depression**		
Condition (level of moderator— organizational trust)	Indirect effect (SE)	Boot 95% CI
Low organizational trust (Mean – 1 SD)	−0.068 (0.069)	−0.200, 0.080
Average organizational trust (Mean)	0.079 (0.044)	0.001, 0.176
High organizational trust (Mean + 1 SD)	0.226 (0.068)	0.097, 0.366
**Index of moderated mediation**		
	Index	Boot SE	Boot 95% CI
Organizational trust	0.198	0.071	0.051, 0.334

## Discussion

This study investigated the mediating effect of resilience and the moderated mediating effect of organizational trust on the effect of nurses' work experiences on depression. The findings are worth consideration.

First, although the score for the organizational trust of nurses could not be compared with other research because none have been conducted using the same tool for nurses, the organizational trust score of 3.03 in this study indicated an intermediate level of organizational trust, based on a scale of five points. The resilience score of nurses was 2.15, which was similar to that found in previous studies. Park and Lee (29) surveyed nurses in intensive care units and found a resilience score of 2.12. Jeong and Shin (30) also conducted a survey on university hospital nurses and found that their resilience score was 2.10. Regarding depression among nurses, 54.5% of the nurses experienced depression, while 65.6% of those with experience of working in a COVID-19 unit experienced depression; from these 35.9% had depression above a moderate level, indicating that the level of depression of nurses needs to be addressed.

Second, nurses' work experiences in a COVID-19 unit directly affect resilience and depression and indirectly affect depression through resilience. In particular, nurses' work experiences in a COVID-19 unit have a negative correlation with resilience and a positive correlation with depression, which implies that their work experiences in a COVID-19 unit lower their resilience and increase their depression. In a previous study, 50.4% of healthcare professionals providing care to COVID-19 patients in China (31) and 43.6% of nurses in an emergency room experienced depression, and 16% of the nurses in the emergency room experienced moderate or higher levels of depression (32). This is because they worked on the front line for long hours, providing care to patients diagnosed with highly infectious diseases and because their psychological burden was higher than that of physicians (33). For these reasons, a work environment characterized by a higher level of work intensity, excessive workload, and insufficient compensation (32, 34) was identified as featuring relevant factors that increase the risk of depression. As shown in this study, the prevalence of depression among nurses in relation to their work environment increased during the COVID-19 pandemic. Hospitals should therefore prepare substantial work environment strategies to mitigate nurses' depression.

With regard to the relationship between work experiences in a COVID-19 unit and resilience, this study showed that nurses' work experiences in a COVID-19 unit decreased their resilience. Previous research has reported that people exhibited resilience after a traumatic event (13), in contrast to the findings of this study. The reason for this may be that the working environment, which was not systematically equipped with workforce support and compensation (2, 31), was affected and that the research was conducted when the end of the COVID-19 pandemic had not been projected. Thus, it may be that the nurses' low resilience caused by this chaotic work environment resulted in depression. In addition, the findings of this study showed that the indirect effect of nurses' work experiences on depression via the mediating factor of resilience was greater than its indirect effect. This suggests that there is a need not only to improve nurses' work environment in COVID-19 units but also to develop nurses' resilience through nurse-focused support.

Third, the resilience of nurses with high organizational trust was significantly lowered by work experiences in a COVID-19 unit. These results are similar to those of a previous study, which found that during traumatic events, the heavy workload for nurses with high trust in their organization resulted in lower resilience, for which they blamed themselves (9). The ever-increasing number of confirmed and suspected cases, the lack of PPE, and feelings of inadequate support place a psychological burden on healthcare professionals (31). In other words, this study revealed that nurses showed low resilience be-cause the organization did not respond and support them to the extent that the nurses could trust.

Fourth, organizational trust had a direct effect on resilience, showing a positive correlation, and a moderating effect between nurses' work experiences in a COVID-19 unit and resilience. As described above, nurses with work experiences in a COVID-19 unit showed low resilience when their organizational trust was high. However, as a result of the moderated mediating effect, organizational trust lowered depression by reducing the effect of work experiences in a COVID-19 unit on resilience; hence, it is necessary to develop and apply strategies to increase nurses' organizational trust. Considering the finding that 75.8% of the nurses in this study reported that they would not apply to work in a COVID-19 unit, future research should focus on the specific support needed for nurses at an organizational level to increase their organizational trust.

### Strengths and Limitations

The strength of this study lies in the fact that the data were collected after the threat of the second wave of COVID-19 loomed in South Korea. The data reflected the levels of work experience, organizational trust, resilience, and depression among the nursing staff under the circumstances confronting them prior to the expected rapid increase in the number of infected cases. Hence, the findings of the data analysis provide representative evidence from nurses for hospital administrators.

This study had some limitations. First, the study used a convenience sampling method in a general hospital with over 500 beds to recruit participants, inducing a problem with the generalization of the findings to other middle-level or tertiary hospitals with over 1,000 beds. Second, the questionnaire used in this study was based on a self-reported survey. The participants' perceived resilience and depression may be overestimated or underestimated; hence, interpretation of the results of this study should be conducted with caution. Third, this study did not consider the impact of daily patient census on nurses' resilience and depression. It should be included as a variable in the future study.

### Implications for Nursing Management

During the COVID-19 pandemic, the prevalence of depression among nurses affected by their work environment has increased, and hospitals need to prepare substantial improvement strategies for the work environment to mitigate nurses' depression. Additionally, the findings of this study showed that nurses' work experiences with higher levels of nurses' resilience had a significant impact on depression. Thus, besides improving nurses' work environment in COVID-19 units, there is a need to develop nurses' resilience through nurse-focused support. Lastly, healthcare organizations should provide organizational support to nurses so that the organizational trust and resilience of nurses can be maximized.

## Conclusions

This study aims to provide practical data for preparing organizational systems to reduce depression among nurses working in hospitals during the COVID-19 pandemic by identifying the mediating effects of resilience and the moderated mediating effects of organizational trust on the impact of nurses' COVID-19 unit work experiences on depression. This study is valuable for future pandemic preparedness because it highlights the role of healthcare organizations in protecting nursing staff resilience and preventing depression. Healthcare organizations need to establish a system to improve nurses' resilience and organizational trust if they have not already done so.

## Data Availability Statement

The datasets presented in this article are not readily available because they are covered by data protection for sensitive and identifiable data, further inquiries can be directed to the corresponding author(s).

## Author Contributions

E-YD and SC: conceptualization, validation, writing—original draft preparation, writing—review and editing, visualization, and supervision. E-YD: methodology, software, formal analysis, investigation, resources, data curation, and project administration. Both authors have read and agreed to the published version of the manuscript.

## Conflict of Interest

The authors declare that the research was conducted in the absence of any commercial or financial relationships that could be construed as a potential conflict of interest.

## Publisher's Note

All claims expressed in this article are solely those of the authors and do not necessarily represent those of their affiliated organizations, or those of the publisher, the editors and the reviewers. Any product that may be evaluated in this article, or claim that may be made by its manufacturer, is not guaranteed or endorsed by the publisher.
